# Warfarin maintenance dose Prediction for Patients undergoing heart valve replacement— a hybrid model with genetic algorithm and Back-Propagation neural network

**DOI:** 10.1038/s41598-018-27772-9

**Published:** 2018-06-26

**Authors:** Qian Li, Huan Tao, Jing Wang, Qin Zhou, Jie Chen, Wen Zhe Qin, Li Dong, Bo Fu, Jiang Long Hou, Jin Chen, Wei-Hong Zhang

**Affiliations:** 1 Department of Evidence-based Medicine and clinical epidemiology, West China Medical School of Medicine/West China Hospital, Sichuan University, Chengdu, China; 2 Department of Career development, The fourth affiliated hospital of Anhui Medical University, Hefei, China; 3 Department of Nutrition, The Second Affiliated Hospital of Chongqing Medical University, Chongqing, China; 4 Department of Anesthesiology, China Mianyang Central Hospital, Mianyang, China; 5 Department of Social Medicine and Health Management, Shandong University, Jinnan, China; 6 Department of Cardiovascular Surgery, West China Hospital, Sichuan University, Chengdu, China; 7 Department of Cardiovascular Surgery, Tianjin central hospital, Tianjin, China; 8 Department of Research Laboratory for Human Reproduction, Faculty of Medicine, Université Libre de Bruxelles (ULB), Bruxelles, Belgium; 9 International Centre for Reproductive Health (ICRH), Ghent University, Ghent, Belgium; 10 Epidemiology, Biostatistics and Clinical Research Centre, School of Public Health, Université Libre de Bruxelles (ULB), Bruxelles, Belgium

## Abstract

Warfarin is the most recommended anticoagulant drug for patients undergoing heart valve replacement. However, due to the narrow therapeutic window and individual dose, the use of warfarin needs more advanced technology. We used the data collected from a multi-central registered clinical system all over China about the patients who have undergone heart valve replacement, subsequently divided into three groups (training group: 10673 cases; internal validation group: 3558 cases; external validation group: 1463 cases) in order to construct a hybrid model with genetic algorithm and Back-Propagation neural network (BP-GA), For testing the model’s prediction accuracy, we used Mean absolute error (MAE), Root mean squared error (RMSE) and the ideal predicted percentage of total and dose subgroups. In results, whether in internal or in external validation group, the total ideal predicted percentage was over 58% while the intermediate dose subgroup manifested the best. Moreover, it showed higher prediction accuracy, lower MAE value and lower RMSE value in the external validation group than that in the internal validation group (p < 0.05). In conclusion, BP-GA model is promising to predict warfarin maintenance dose.

## Introduction

Warfarin is a commonly used oral anticoagulant in heart valve replacement^1^. However, warfarin has the following three limitations: the first is the narrow therapeutic window, which means that the effective dose is very close to the threshold dose and even a small dose variation can cause serious bleeding events; the second is the obvious variation of individual dose. To be specific, the difference in the individual warfarin dose can be as high as 20 times^2^, meanwhile, the variation on the individualized warfarin clearance rate can reach to 50%^3^; the third is that dose management is affected by a variety of factors, such as age, race, drug combination, dietary intake, and gene polymorphisms of CYP2C9 and VKORC1^4^, and a complex nonlinear relationship between these factors and warfarin dose might be existed^5^. Hence, the accuracy of warfarin dose is critical to the safety and effectiveness, and it is significant to individualize warfarin treatment. In addition, it is a challenge for physicians to apply the individual warfarin treatment with high accuracy based only on their clinical experience.

In plenty of warfarin dose prediction models^5–9^, MLR model is the most common one which manifests superior accuracy. However, the complex nonlinear relationship between the factors mentioned above and warfarin dose made the MLR model an inappropriate method that can predict warfarin maintenance dose accurately^10^. Hence, a more appropriate model is in need to optimize individual treatment. With the rapid development of artificial intelligence technology, which can utilize dataset to construct and address nonlinear model through complicated connection^11,12^, it is possible to find a more appropriate algorithm than MLR. BP-GA, a hybrid artificial intelligent algorithm with genetic algorithm and Back-Propagation (BP) neural network, may be exactly what we look for. As it combines the benefit of the two algorithms, which can identify nonlinear relationships by its adaptive learning features^13–15^, in the meantime, it is not easy to fall into locally optimal solution^16^.

Accordingly, this study was aimed to construct the BP-GA model to predict individual warfarin maintenance dose and to evaluate its prediction accuracy.

## Material and Methods

The protocol of this study has been approved by the Ethics Committee of West China Hospital of Sichuan University (ChiECRCT-201792). For that this was a retrospective study, in the Ethical approval documents, the informed consent has been exempted. The methods were carried out in accordance with the relevant guidelines and regulations.

### Participants

The participants were patients undergoing heart valve replacement extracted from the database “Chinese Low Intensity Anticoagulant Therapy after Heart Valve Replacement” (CLIATHVR), which was collected from April 1st, 2011 to December 31st, 2015, through a multi-central registered clinical system in 35 medical centers all over China.

The inclusion criteria: (1) Chinese people; (2) age over 18 years; (3) receiving warfarin as the only oral anticoagulation in regular and monitor by INR as index after receiving heart valve replacement; (4) assuring the fluctuation of INR less than 0.2 units for three times continuously and the INR range was 1.5–2.5 during the later follow-up. The included patients should meet all the above criteria.

The exclusion criteria: (1) severe liver or kidney dysfunction before or after the operation; (2) drug combination of non-steroidal anti-inflammatory drugs or other drugs affecting anticoagulation effect; (3) anticoagulant complications (thrombosis; embolism; bleeding; death) occurred during anticoagulant therapy (considering our objective was to predict warfarin maintenance dose. In that dose, it realized the ideal state where warfarin took great anticoagulation effect and complications did not occur). The patient who was in any of the above situations would be excluded.

### Included Variables

#### The input variables

The input variables were extracted by two methods: the analysis of covariance and the enrollment of mandatory variables.

Such specific steps were performed: firstly, for data cleaning, based on the clinical professional knowledge, we screened items from all the 706 items included in the database as the latent input variables. Then the analysis of covariance was used for extracting the primary input variables that have statistical significance (Type I, α = 0.05). Finally, we enrolled in the mandatory variables relevant to warfarin in clinic whether it had statistical difference or not.

#### The output variable

Warfarin maintenance dose was the output variable, which was identified when the INR value was all at the target range of 1.5–2.5 and the fluctuation was less than 0.2 units for three times in succession.

### Data set

We divided the eligible cases into three groups: Group A (Training group), Group B (the internal validation group) and Group C (the external validation group).

According to the distribution method mentioned by Steyerberg^17^ and Lópe^18^, we chose the medical centers enrolling less than 200 cases (the medical centers of small cases size often do not belong to professional cardiothoracic hospital or Tertiary hospital, and have low compatibility) as Group C. The remaining was randomly divided into group A and group B by the ratio of 3:1. Group A was used to generate model, Group B and C were used to verify the predication accuracy of internal and external validation, respectively.

### Model construction

#### The introduction of BP-GA model

**Figure 1:**
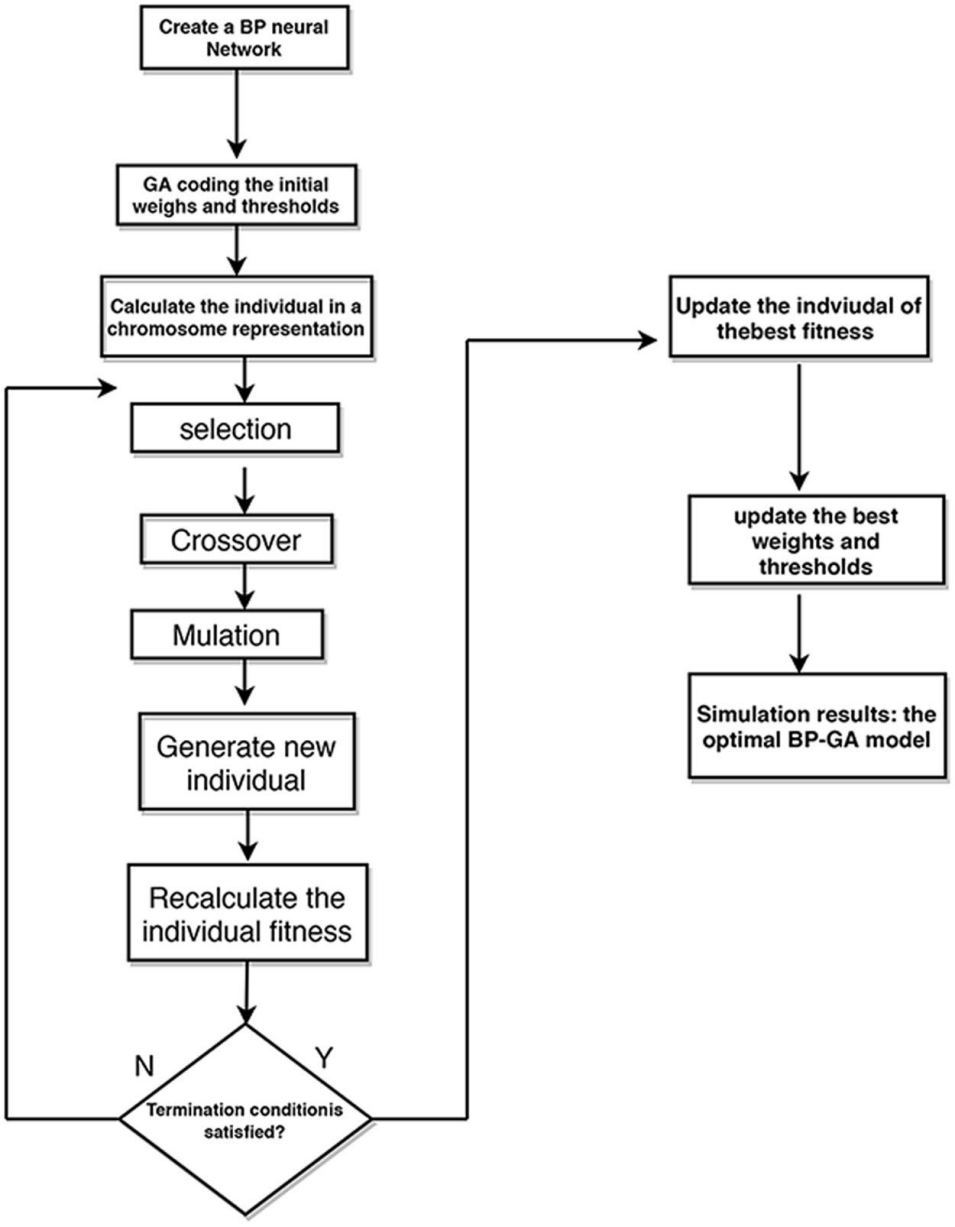
The flow chart of BP-GA model construction.

The BP neural network of three layers (input layer, hidden layer and output layer) has been widely used in medicine with superior solution of nonlinear relationship and good error-tolerance capability. However, BP neural network is easy to fall into the local optimal solution, and the convergence performance of BP neural network is weak^19,20^. GA (genetic algorithm) follows the principle of evolution and takes the individual of good evolution as the optimal solution through searching the whole solution space. Hence, it can obtain the global optimal solution to optimal BP neural network. In our study, the process to construct BP-GA Model included two basic parts as depicted in Figure 1.

#### Part one: BP neural network modeling

It was a process of information transfer in feed-forward and error back propagation. To be specific, according to the given input and output layer sample data, it went on training to construct the network structure in the propagation process until the error between actual output and target output met minimum. Accordingly, in our study, the final independent variables were the neurons in the input layer, and the neuron in the output layer was warfarin maintenance dose. The number of neurons in hidden layer was in the range of $\sqrt{{\rm{m}}+{\rm{n}}}+{\rm{\alpha }}$ (α was the constant integer of 1–10, m and n were the numbers of neurons in the input layer and output layer, respectively.), which was determined when the error between actual output and target output got the minimum. The error was the value of MAE (the Mean absolute error between the predicted dose and the actual dose). The smaller the MAE value was, the more accurate the prediction model was.

#### Part two: The weights and thresholds optimization by GA algorithm

Although the network structure has been constructed, it generated the initial weights and thresholds randomly. The improper initial weights and thresholds would worsen the prediction accuracy. Hence, it was inevitable to go on weights and thresholds optimization by GA algorithm.

#### The process of optimization was listed as the following steps

Step 1: A group of individuals composed of a certain generation, each individual was described as a chromosome. Chromosome consisted of a series of real numbers, which represented the connection weights between hidden layer and input layer, the connection weights between output layer and hidden layer, and the thresholds in the hidden and output layer.

Step 2: The reciprocal of the absolute value, which represented the difference between the predicted and actual outputs of each individual, was regarded as the fitness function. As shown in eq. (1), it was in direct proportion to the viability of the chromosome.

1 $${{\rm{F}}}_{i}=1/(\sum _{i=1}^{n}abs({y}_{i}-{o}_{i}))$$

(n denotes the number of neurons in the output layer, *y*_*i*_ and *o*_*i*_ represent the predicted and actual outputs of the *i*th neuron, respectively).

Step 3: The weights and the thresholds optimization can be obtained by performing the following operations, such as selection, crossover, and mutation.

1. The roulette method was used as the selection strategy according to a certain probability (P*i*) based on the size of fitness value, as showed in eq. (2).
  2 $${\rm{p}}i=\frac{{{\rm{f}}}_{i}}{{\sum }_{i=1}^{N}{{\rm{f}}}_{i}}$$
  (f*i* denotes the fitness value of the *i*th individual, N denotes the number of population. The larger the value of individual fitness is, the greater the opportunity to be selected will be).
2. The crossover operation was that the two individuals were selected from the group according to a given probability, and the parts of the two individual’s codes were exchanged to get two new individuals.
3. The mutation operation was that individuals can be selected according to a given mutation probability. The chromosome mutation position of the individual was randomly determined.

Next, it went on the process of iterative evolution from step 1 to step 3 until getting the Near-Optimal fitness value or going to the default maximum generation of evolution. The output of this process was the individual with the best fitness, and the individual consists of the weights and threshold, which would be used as the final weights and thresholds of the BPNN.

#### The parameters and software used in our study

The BP neural network was constructed by Neural Network Toolbox of MATLAB R2010b. The parameters of BP neural network are set according to the engineering experiences, in our study, they were listed as follows: the training times were 1000, the target of error was 0.001 and the learning rate was 0.1.

The GA was constructed by the GAOT Toolbox in MATLAB R2010b. The parameters were set as follows: The size of population was 50, the generation of evolution was 100, the crossover rate was 0.95, and the mutation rate was 0.09.

### Model validation

We used MAE, Root mean squared error (RMSE: the square root of the mean square error between the predicted dose and actual dose) to measure the prediction accuracy. In the meantime, according to the defined method of Klein *et al*.^7^, the ideal predicted percentage (the percentage of whose absolute error between the predicted dose and actual dose was within 20% of the actual dose) was used to test the clinical utility of BP-GA. The smaller the value of MAE and RSME was, the better the prediction accuracy was. And the larger the ideal predicted dose percentage was, the better the clinical utility was.

Dose subgroups analysis was also conducted to decrease the clinical heterogeneity, which was based on the 25% and 75% quartile of the actual value of warfarin maintenance dose: high dose >3.0 mg/d, intermediate dose 2.5 mg/d–3.0 mg/d, low dose <2.5 mg/d.

### Statistical analysis

The independent sample t-test was used for assessing the statistical difference between two groups including training and internal validation groups; training and external validation groups, internal validation and external validation groups. Difference of the predicted percentage between the internal and external validation was analyzed by chi-square test, and the statistical significance level of all analysis was set up as 0.05 with two-sided test by SPSS 20.0.

## Results

### Participants’ characteristics

**Figure 2:**
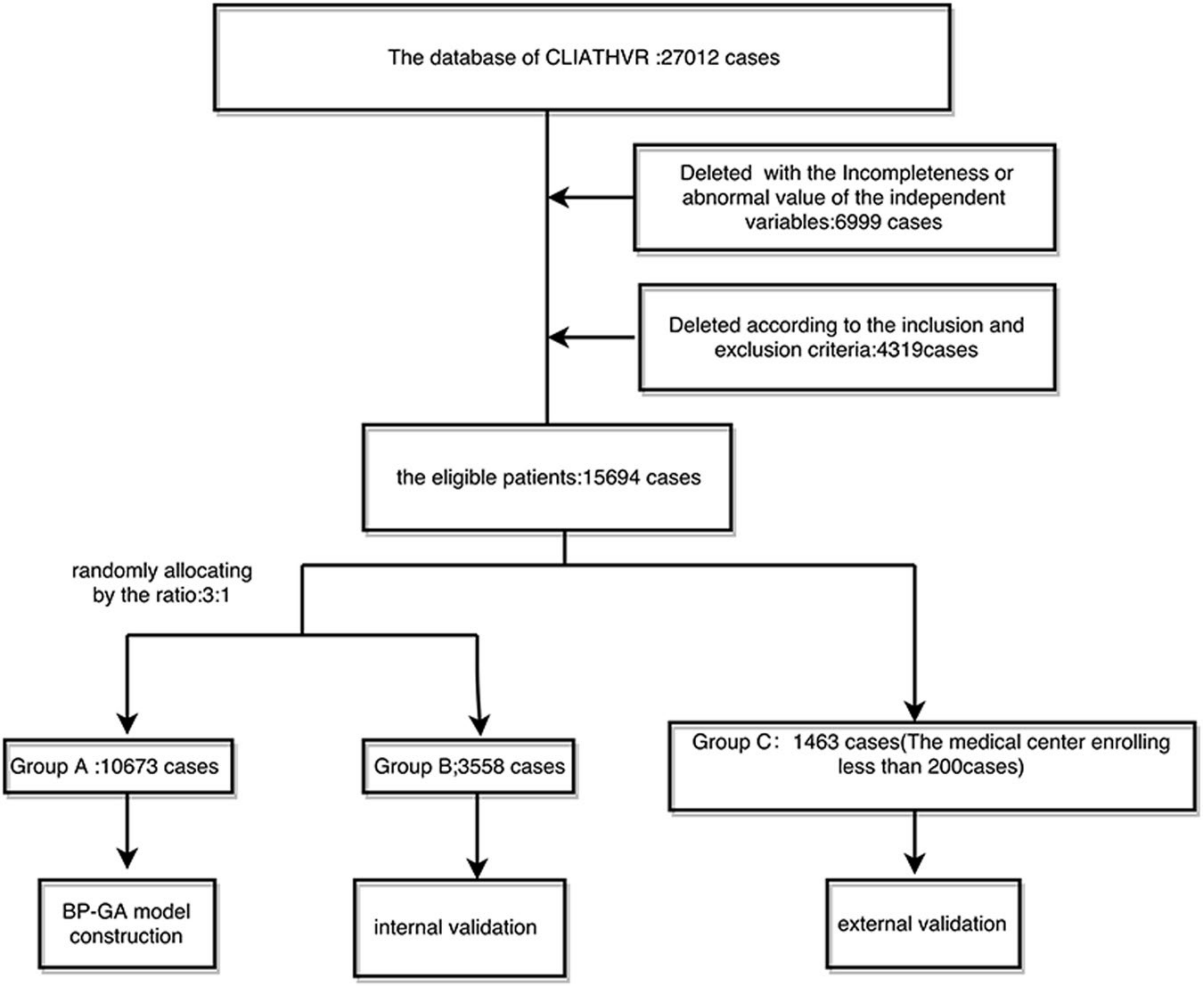
The flow diagram of data sets.

As the flow diagram showed in Figure 2, we finally included15694 eligible cases in the analysis, the cases in training group, internal validation group and external validation group were 10673, 3558 and 1463, respectively. The basic characteristics of the participants were showed in Table 1. Overall, the patients’ age was centered on 40–65 years old (the mean age = 50.24 years). The male and female sexed ratio was near to 9:11. Most eligible patients were Han-Chinese, and the mean warfarin maintenance dose was 2.73 ± 0.73 mg/d.

**Table 1:** The basic characteristic of the whole study.

Characteristic (unit)	Total cases	Training group	internal validation	external validation
	(n = 15694)	(n = 10673)	(n = 3558)	(n = 1463)
	N (%)/(X ± S)	N (%)/(X ± S)	N (%)/(X ± S)	N (%)/(X ± S)
Age (year)	50.24 ± 11.17	50.43 ± 11.12	50.07 ± 11.19	49.35 ± 11.48*
Height (cm)	162.90 ± 8.18	162.87 ± 8.19	163.12 ± 8.22	162.52 ± 7.96
Weight (kg)	60.77 ± 10.99	60.89 ± 11.06	60.79 ± 10.83	59.85 ± 10.77*
BSA (m^2^)	1.62 ± 0.18	1.62 ± 0.18	1.62 ± 0.17	1.60 ± 0.17*
Gender (Male)	7142 (45.5)	4853 (45.5)	1652 (46.4)	637 (43.5)
Nationality (han)	15003 (95.6)	10209 (95.7)	3407 (95.8)	1378 (94.8)
Hypertension history	1748 (11.1)	1210 (11.3)	399 (11.2)	139 (9.5)
Operation history	1702 (10.8)	1164 (10.9)	389 (10.9)	149 (10.2)
EF (%)	58.36 ± 8.79	58.26 ± 8.80	58.52 ± 8.61	58.71 ± 9.13
Left ventricular diastolic diameter (mm)	57.38 ± 14.44	57.65 ± 14.63	57.43 ± 14.18	55.27 ± 13.48*
Inner diameter of left atrium (mm)	50.15 ± 14.06	50.20 ± 13.74	49.67 ± 14.09	50.98 ± 16.11
Inner diameter of right atrium (mm)	38.84 ± 14.01	38.69 ± 13.81	38.33 ± 13.65	41.20 ± 16.00*
ALT (IU/L)	26 ± 20.29	26.03 ± 20.36	26.27 ± 20.42	25.16 ± 19.35
AST (IU/L)	26.50 ± 16.38	26.46 ± 16.26	26.43 ± 17.01	26.94 ± 15.66
Total albumen (g/L)	68.51 ± 6.77	68.48 ± 6.71	68.43 ± 6.80	68.88 ± 7.07*
Albumin (g/L)	41.60 ± 4.70	41.62 ± 4.66	41.66 ± 4.64	41.24 ± 5.07*
ALB/GLB	1.62 ± 0.45	1.62 ± 0.46	1.63 ± 0.43	1.57 ± 0.47
Urea nitrogen (mmol/L)	6.13 ± 2.08	6.13 ± 2.07	6.13 ± 2.11	6.16 ± 2.08
Creatnine (umol/L)	78.58 ± 20.44	79.00 ± 20.39	78.05 ± 20.48	76.79 ± 20.57*
Preoperative PT of one day before valve replacement (s)	13.22 ± 3.61	13.23 ± 3.69	13.13 ± 3.19	13.39 ± 4.03
preoperative APTT of one day before valve replacement (s)	31.64 ± 8.30	31.43 ± 8.03	31.13 ± 7.96	34.02 ± 10.40*
Preoperative INR	1.12 ± 0.46	1.12 ± 0.46	1.12 ± 0.52	1.12 ± 0.35
**NYHA classification**				
I class	184 (1.2)	130 (1.2)	45 (1.3)	9 (0.6)
II class	3646 (23.2)	2480 (23.2)	808 (22.7)	358 (24.5)
III class	11323 (72.1)	7687 (72.0)	2601 (73.1)	1035 (70.7)
IV class	541 (3.4)	376 (3.5)	104 (2.9)	61 (4.2)
Mitral valve surgery (replacement)	11241 (71.6)	7624 (71.4)	2524 (70.9)	1093 (74.7)*
TricuspidValvesurgery (replacement)	6338 (40.4)	4314 (40.4)	1461 (41.1)	563 (38.5)
Aortic valve surgery (replacement)	8385 (53.4)	5715 (53.5)	1942 (54.6)	728 (49.8)*
Pulmonary artery surgery (replacement)	37 (0.2)	20 (0.2)	11 (0.3)	6 (0.4)
Left atrial appendage occlusion (treatment)	1177 (7.5)	766 (7.2)	249 (7.0)	162 (11.1)*
Thrombus removal	1197 (7.6)	799 (7.5)	269 (7.6)	129 (8.8)
Radiofrequency ablation	1299 (8.3)	871 (8.2)	310 (8.7)	118 (8.1)
Origin of warfarin (made in China)	7733 (49.3)	5433 (50.9)	1760 (49.5)	540 (36.9)*
The time of first anticoagulant (n days after surgery)	1.88 ± 1.5	1.88 ± 1.06	1.87 ± 1.05	1.92 ± 1.01
Maintenance dose at discharge time (mg/d)	2.73 ± 0.73	2.73 ± 0.74	2.75 ± 0.74	2.69 ± 0.70
**Warfarin dose-subgroup**				
Low-dose	2117 (13.5)	1454 (13.6)	465 (13.1)	198 (13.5)
intermediate-dose	11848 (75.5)	8077 (75.7)	2678 (75.3)	1093 (74.7)
High-dose steady-state INR	1729 (11.0) 2.06 ± 0.18	1142 (10.7) 1.92 ± 0.17	415 (11.7) 1.89 ± 0.19	172 (11.8) 2.15 ± 0.15*

Note: *P < 0.05 (independent sample t-test of a certain characteristic between the external validation and the training group); Han = 0, all ethnic = 1; BSA: Body surface area = 0.0061 × height (cm) + 0.0128 × weight (kg) − 0.1529; EF: Ejection fraction; LVDD: Left ventricular end diastolic dimension, LAD: Left atrial diameter; RAD: Right atrial diameter; ALT: Alanine transaminase; AST: Aspartate aminotransferase; APTT: Activated partial thromboplastin time; INR: International normalized ratio.

There was no statistical difference (p > 0.05) between the training group and the internal evaluation group in characteristics. And there was difference (p < 0.05) between the training and the external evaluation group in relevant demographic and clinical features such as age, weight. Compared with the internal validation group, the patients in the external validation group have statistical difference (P < 0.05) in many characteristics, such as weight, BSA (Body surface area), APTT (Activated partial thromboplastin time) and steady-state INR.

### Included variables

#### The independent variables

Firstly, in the process of data cleaning, 45 items were screened as the latent independent variables after removing the items not related to warfarin dose or whose integrity was under 50%.

Then, the primary 10 input variables were selected by the analysis of covariance (η^2^ ≥ 0.01 and p < 0.05) in Table 2, η^2^ meant the contribution of a certain input variable on the output variable. Considering the requirement of model conciseness, η^2^ was set as more than 0.001. The 10 input variables were age, EF (Ejection fraction), left ventricular diastolic diameter, operation history), albumin, urea nitrogen, creatinine, preoperative APTT of one day before valve replacement, timing of first anticoagulant and warfarin origin. Operation history meant whether the included patient has undergone other surgery before heart valve replacement, and warfarin origin meant where the warfarin is made, in China or abroad. We used 1 to represent warfarin made in China (Qilu pharma or Shanghai Xinyi Pharma) and 2 to represent warfarin made abroad (Orion Corporation Orion Pharma).

**Table 2:** Variables after screened by the analysis of covariance.

Included variables (unit)	partial *η*^2^
Age (year)	0.002
EF (%)	0.001
Left ventricular diastolic diameter (mm)	0.001
Operation history	0.001
Albumin (g/L)	0.001
Urea nitrogen (mmol/L)	0.001
Creatinine (umol/L)	0.003
preoperative APTT of one day before valve replacement (s)	0.004
Warfarin origin	0.66
The time of first anticoagulant (d)	0.06

Note: APTT: Activated partial thromboplastin time.

EF: Ejection fraction.

Operation history: it is a categorical variable and means whether the included patient has done other surgery before heart valve replacement, we used 1 to represent patient not having done other surgery and 2 to represent patient having done other surgery.

Warfarin origin: it is also a categorical variable and means where the warfarin is made in, China or abroad, we used 1 to represent warfarin made in China and 2 to represent warfarin made abroad.

Next, height and weight were used as the mandatory variables part. The reason was that Gu *et al*.^21^ found the three variables (age, weight and height) can explain 76.8% of the total warfarin dose variation Hence, in the end, 12 independent variables were filtered out.

The output variable: warfarin maintenance dose was the output variable.

### Model construction

**Figure 3:**
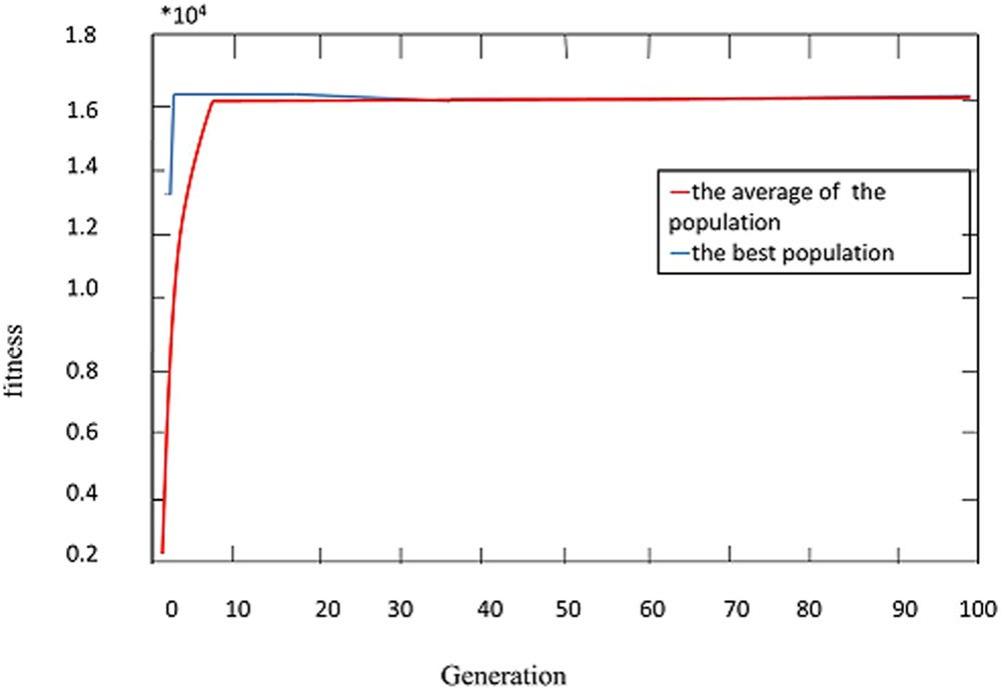
The fitness curve of BP-GA.

**Figure 4:**
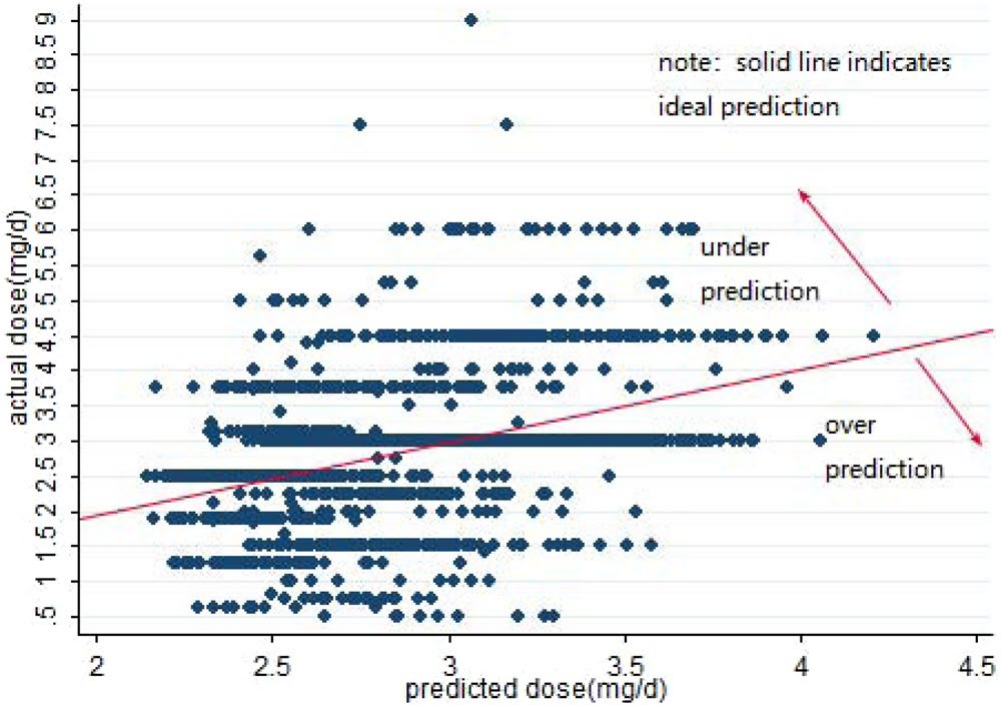
Predicted vs. actual warfarin maintenance dose in the internal validation.

**Figure 5:**
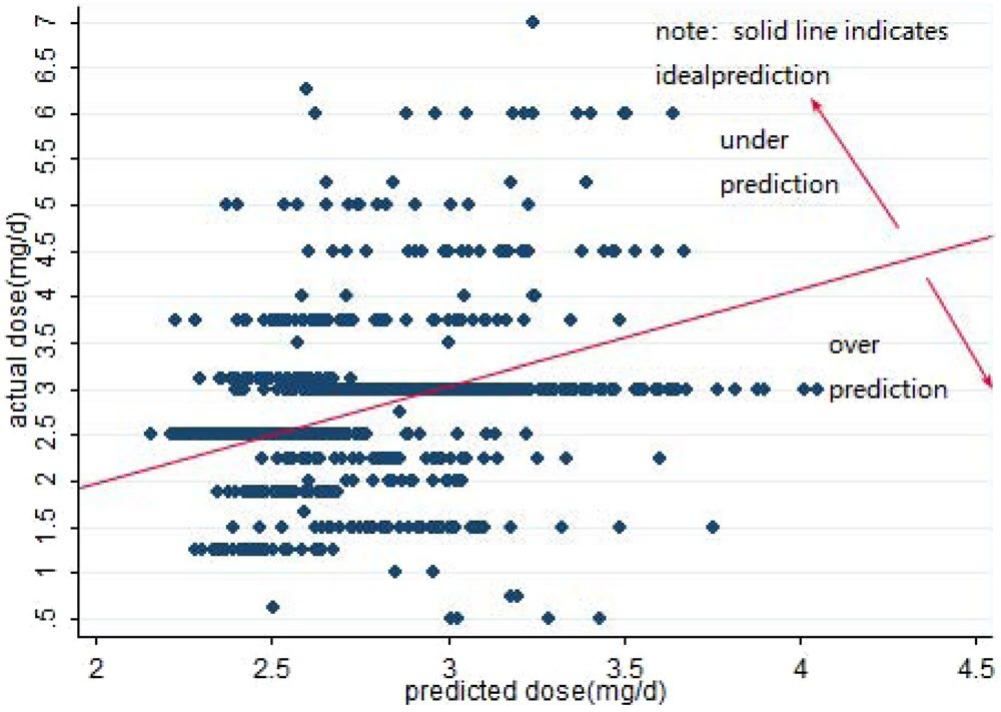
Predicted vs. actual warfarin maintenance dose in the external validation.

The primary BP neural network was as the following: m was 12 because we finally selected 12 input variables, and n was 1 because the output variable only included warfarin maintenance dose, the number of point of hidden layer was 9, which got the minimum value of MAE. As it showed in Figure 3, the whole process of BP-GA model went on the 23rd generation training to get the best and stable value of the fitness, and showed the genetic algorithm can be used for optimizing the weighs and thresholds value. The predicted diagrams were showed in Figures 4 and 5.

### Model validation

In the analysis of the total ideal predicted percentage (Table 3), BP-GA both showed over 58% predicted percentage. Moreover, it showed higher prediction accuracy (p < 0.05) in the external validation group than that in the internal validation group. When considering the MAE and RMSE, the value of MAE was also lower in the external group (internal group: 0.383 mg/d; external group: 0.370 mg/d) with statistical difference (p < 0.05). Meanwhile, the value of RMSE in the external group was lower than that in the internal group (internal group: 0.664 mg/d; external group: 0.656 mg/d).

**Table 3:** The Comparison of total predication accuracy of the BP-GA.

Model	MAE*	RMSE*	Predicted percentage		
			Underestimate (%)	Ideal (%)**	Overestimate (%)
BP-GAiv	0.383 (0.365~0.401)	0.664 (0.631~0.696)	746 (21.0)	2088 (58.7)	724 (20.3)
BP-GAv	0.370 (0.342~0.397)	0.656 (0.606~0.704)	257 (17.6)	920 (62.9)	286 (19.5)

Note: BP-GAiv: the internal validation group.

BP-GAev: the external validation group.

*P < 0.05 (independent sample t-test of the MAE and RMSE between the internal and external validation group, respectively).

**P < 0.05 (chi-square test the ideal percentage between the internal and external validation group).

Ideal: the percentage of patients whose predicted absolute error between predicted dose and actual dose was within 20% of actual dose.

Underestimate: the percentage of patients whose predicted dose was less than actual dose and the predicted absolute error between predicted dose and actual dose was more than 20% of the actual dose.

Overestimate: the percentage of patients whose predicted dose was more than actual dose and the predicted absolute error between predicted dose and actual dose was more than 20% of the actual dose.

In the dose subgroup analysis (Table 4), whether in the internal or external validation, BP-GA had the best predicted percentage in the intermediate dose subgroup. Meanwhile, the predicted percentage of the external group in the intermediate group was higher than (p < 0.05) that in the internal group (the internal group: 77.90%;the external group: 84.20%). What’s more, whether in internal or external validation subgroup, BP-GA model showed over 98% over-prediction in low dose subgroup, and it manifested over 98% under-prediction in high dose subgroup.

**Table 4:** Comparison of the models’ predicated percentage of dose subgroup

Model	Low dose subgroup < = 2.5 mg/d			Intermediate dose subgroup 2.5~3.0 mg/d			High dose subgroup > = 3.0 mg/d		
	Underestimate (%)	Ideal (%)	Overestimate (%)	Underestimate (%)	Ideal** (%)	Overestimate (%)	Underestimate (%)	Ideal (%)	Overestimate (%)
BP-GA iv	0 (0.0)	1 (0.2)	464 (99.8)	334 (12.4)	2085 (77.9)	259 (9.7)	412 (99.3)	2 (0.5)	1 (0.2)
BP-GA ev	0 (0.0)	0 (0.0)	198 (100)	85 (7.8)	920 (84.2)	88 (8.1)	172 (100)	0 (0.0)	0 (0.0)

Note: BP-GA iv: the internal validation group; BP-GA ev: the external validation group.

**P < 0.05 (chi-square test the ideal percentage between the internal and external validation group).

Ideal: the percentage of patients whose predicted absolute error between predicted dose and actual dose was within 20% of actual dose.

Underestimate: the percentage of patients whose predicted dose was less than actual dose and the predicted absolute error between predicted dose and actual dose was more than 20% of the actual dose.

Overestimate: the percentage of patients whose predicted dose was more than actual dose and the predicted absolute error between predicted dose and actual dose was more than 20% of the actual dose.

## Discussion

### The summary of main results

Our study has three important features: firstly, our study was based on a clinical registered system of 27012 cases using warfarin after heart valve replacement; secondly, we used BP-GA, an artificial intelligence method, to build a model based on 15694 eligible patients from the database; thirdly, the average warfarin maintenance dose was 2.73 mg/d, which was less than the previous IWPC^7^ maintenance dose of 4 mg/d. And the target INR value range^22^ was 1.5–2.5, which was less than the western standard (INR 2.0–3.0)^23^. These features proved that Chinese people were more sensitive to warfarin and they should be given low-intensity anticoagulation.

In summary, there was statistical difference (p < 0.05) between training and external evaluation groups, internal and external validation groups, which manifested the two groups were from different samples of divergent demographic and clinical characteristics. When considering the value of MAE, RMSE and total ideal predicated percentage, BP-GA model all showed significant prediction accuracy no matter in internal or external validation. Furthermore, in the dose subgroup, BP-GA model showed the best prediction accuracy in the intermediate dose subgroup. And the prediction accuracy in the external validation was higher than that in the internal validation, which enlightened that BP-GA was a useful model with high external validity.

### The plausibility of final independent variables

In this study, we used two ways (the analysis of covariance and the enrollment of mandatory variables)to select the final independent variables: Hence, in the end, 12 independent variables (age, EF, left ventricular diastolic diameter, operation history, albumin, urea nitrogen, creatinine, preoperative APTT of one day before valve replacement, timing of first anticoagulant, warfarin origin, weight and height) were selected. It has been validated in the previous study^21^ that the three variables (age, weight and height) can explain 76.8% of the total warfarin dose variation. Masayasu *et al*.^24^ found that EF and left ventricular diastolic diameter were related to the formation of thrombus, thus, they are also related to the use of warfarin. Meanwhile, creatinine and urea nitrogen are the typical Laboratory inspection indicators of kidney function. Nita *et al*.^25^ found kidney function influenced warfarin responsiveness. Albumin is one of the major carriers proteins in the body and constitutes approximately half of the protein found in blood plasma, Osama *et al*.^26^ found it had one of the protein’s major binding sites “Sudlow I” which included a binding pocket for the drug warfarin (WAR), hence, albumin is also related to the warfarin dose. In our study, APTT was the preoperative APTT of one day before valve replacement. When referring to Kucuk M *et al*.^27^, they found a preoperative low APTT value may be an indicator for thrombosis in patients who have undergone heart surgery, hence, it affected the postoperative anticoagulation. Furthermore, it may also affect the use of warfarin after heart valve replacement. In Dong *et al*.^28,29^, the warfarin maintenance dose made in China was different from the imported brands. In Lip *et al*.^30^, time in therapeutic range and medical history were related to the bleeding event of warfarin, hence, operation history and the time of first anticoagulant may also influence the use of warfarin. Therefore, the final included variables were plausible reasoning.

### The comparison between the existing models

When considering the total predicted percentage, it was 62.8% of the external validation in our BP-GA model, which was higher than that (48.46%) inYu *et al*.^31^. Yu *et al*.^31^ was the appropriate reference for that it also went on external validation of 130 Han-Chinese after heart valve replacements. However, obvious difference existed between our study and Yu *et al*.^31^, which may be exactly the reasons for the diversity of predicted percentage. The first was the inconsistency between the training group and the external validation group. Our training group and the external validation group had the same characteristics of the single disease type (undergoing heart valve replacement), the single ethnic (Chinese) and the same INR target value (1.5–2.5). However, Yu *et al*.^31^ used the existing authoritative IWPC^7^ model, the training group was multi-ethnic and multi- disease with a certain target INR value (2.5–3.0), which was different from its own external validation group. Secondly, it was the different sample size of the training group. Our BP-GA model was constructed by the training group of 10673 cases, which was larger than the training group of IWPC model (4043 cases) in Yu *et al*.^31^. It was in accord with the fact that the lager sample size would achieve better prediction performance^32^. Thirdly, comparing with IWPC model, the BP-GA model of our study had strong generalization ability to address the nonlinear relationship.

When the prediction performance was assessed through MAE, our BP-GA model was less than 0.40 mg/d and better than that of Li *et al*.^9^ (over 0.60 mg/d), it used the seven models (SVR, ANN, RT, MLR, RFR, BRT and MARS) to predict warfarin maintenance dose of 1295 Chinese people. The reasons why our model showed better prediction accuracy may because of larger samples size and a new artificial intelligence model used in our study. However, when comparing with Fu-hua model^33^, which was constructed by the training group of Chinese patients and went on external validation of Chinese patients having undergone heart valve replacements, its MAE (0.13 mg/d) was lower than ours. Maybe the following unique identities of Fu-hua study caused the overestimation of prediction accuracy: firstly, in Fu-hua study, it enrolled in genetic data, which can explain 30~40% of the variation degree of warfarin maintenance dose in individuals^34^; secondly, Fu-hua study was a single center trial, the training group and validation group both came from the same hospital, they had the similar demographic and clinical characteristics. Hence, above all, our BP-GA still achieved high prediction accuracy with comparatively low MAE. When the prediction accuracy was manifested in RMSE, whether in the internal or external validation, the value of RMSE of our BP-GA model was under 0.7 mg/d, in other words, it was approximately under 5.0 mg/week, which was absolutely lower than that of the famous IWPC^7^ clinical model (13.8 mg/week)^35^. IWPC^7^ model gathered 5052 warfarin-treated patients in total, who were from different ethnicities, 21 various research groups, 9 countries, and 4 continents. Because the smaller the value of RMSE is, the more precise the method is. Accordingly, we can see the improvement achieved in prediction accuracy with the use of BP-GA model.

In the dose subgroup analysis, our BP-GA model and the IWPC model in Yu *et al*.^31^ were in line that they both showed best predicted percentage of intermediate-dose subgroup, which had the biggest sample size of the training group. Furthermore, compared with Yu *et al*.^31^, the prediction accuracy of our BP-GA in the intermediate subgroup was better. We may find the reason through the specific case distribution of subgroup. In our study, the proportion of the case in the intermediate dose subgroup (75.7%) was higher than that of IWPC^7^ model (53%). Accordingly, it also explained why our BP-GA model showed weak prediction accuracy of low and high dose group (over-prediction in low dose subgroup and under-prediction in high dose group). To be specific, comparing with the sample size in intermediate dose subgroup, the size in low and high dose subgroup was small. And BP-GA model captured more characteristics of intermediate dose group. As a result, the predicted dose was near to intermediate dose whether in the low or high dose subgroup. Hence, it showed over-prediction in low dose subgroup and under-prediction in high dose group. Because the over-estimation would cause the overdose use of warfarin, which was related to the severe symptoms of bleeding (hematuria; bleeding from mucous membranes of the nose or gums; ecchymosis on the extremities; bleeding from the gastrointestinal tract; massive liver hematoma; diffuse alveolar hemorrhage)^36–40^, and warfarin-related hemorrhages result in thousands of emergency department visits and hospital admissions annually^41^. Meanwhile, under-estimation caused the under-dose use of warfarin, which was in accord with insufficiency of anticoagulation and the presence of thrombosis^42^, a main cause of death and disability worldwide^43^. Hence, in the following study, it is inevitable to find a proper way such as stratified training to improve the prediction accuracy of low and high dose subgroups. And it also reminded us that we had better consider genetic factors when predicting maintenance dose of the low and high dose group, but there was no need in the intermediate dose group of the biggest sample size, which will lessen the medical burden, particularly under the fact that the cost of genotype testing was too expensive and has not been covered by medicine reimbursement in China.

## Limitation and Future

There were some limitations of this study need to be addressed. One limitation was that we did not add the genetic information and obvious influential variables into BP-GA model, such as drug combination and diet, which may influence the prediction accuracy. What’s more, it was a retrospective study, hence, the BP-GA model only described the existed phenomenon. And we may discard some important information when we deleted the items whose integrity was under 50%. Before BP-GA model going to clinical application as a useful prediction model, a prospective study was in need in the following series study to validate its predication accuracy and to improve the integrity of follow-up. Meanwhile, the analysis of covariance and compulsorily enrolling the variables used for selecting variables may leave out some important features having latent non-linear relationship with the outcome and lower the prediction accuracy of BP-GA model. Hence, in the future study, a more appropriate method of variables selection is in need. And in fact, the INR and measurements will vary from day to day from the initiation of therapy, thus, it was better to confirm the INR of a certain day to use as the potential input variable. However, our study was a retrospective study, which was based the existed database. In the original data, the frequency and day to start measure INR after valve replacement was not fixed. Hence, our study was difficult to collect INR of a certain day and we hadn’t tested INR in the variable selection. In the following prospective study, it recommends us to collect INR value of a certain day to improve prediction accuracy.

## Conclusion

In conclusion, BP-GA model was a promising model to predicate warfarin maintenance dose for patients undergoing valve replacement, because in both of the total and dose subgroup analysis, BP-GA all showed high prediction accuracy, particularly in the external validation group which represented the condition of real clinical practice.

## References

[1] Nishimura, R. A. *et al*. AHA/ACC Focused Update of the 2014 AHA/ACC Guideline for the Management of Patients With Valvular Heart Disease. *Journal of the American College of Cardiology* (2017). 10.1016/j.jacc.2014.02.536 24603191

[2] Anderson JL (2007). Randomized trial of genotype-guided versus standard warfarin dosing in patients initiating oral anticoagulation. Circulation.

[3] Weiss P, Halkin H, Almog S (1986). The negative impact of biological variation in the effect and clearance of warfarin on methods for prediction of dose requirements. Thrombosis and haemostasis.

[4] Jonas DE, McLeod HL (2009). Genetic and clinical factors relating to warfarin dosing. Trends in pharmacological sciences.

[5] Zhou Q (2014). Use of artificial neural network to predict warfarin individualized dosage regime in Chinese patients receiving low-intensity anticoagulation after heart valve replacement. International journal of cardiology.

[6] Xu H (2015). Comparison of the Performance of the Warfarin Pharmacogenetics Algorithms in Patients with Surgery of Heart Valve Replacement and Heart Valvuloplasty. Thrombosis research.

[7] Klein TE (2009). Estimation of the Warfarin Dose with Clinical and Pharmacogenetic Data. The New England journal of medicine.

[8] Gage BF (2008). Use of pharmacogenetic and clinical factors to predict the therapeutic dose of warfarin. Clinical pharmacology and therapeutics.

[9] Li X (2015). Comparison of the predictive abilities of pharmacogenetics-based warfarin dosing algorithms using seven mathematical models in Chinese patients. Pharmacogenomics.

[10] Ugrinowitsch C, Fellingham GW, Ricard MD (2004). Limitations of ordinary least squares models in analyzing repeated measures data. Medicine and science in sports and exercise.

[11] James AH, Britt RP (1993). Prospective comparative study of computer programs used for management of warfarin. J Clin Pathol.

[12] Ageno W, Johnson J, Nowacki B, Turpie AG (2000). A computer generated induction system for hospitalized patients starting on oral anticoagulant therapy. Thrombosis and haemostasis.

[13] Cybenko G (1992). Approximation by superpositions of a sigmoidal function. Mathematics of Control, Signals and Systems.

[14] Funahashi K-I (1989). On the approximate realization of continuous mappings by neural networks. Neural Networks.

[15] Lei, W. The Principle, classification and application of artificial neural network. *Science & Technology Information*, 240–241 (2014).

[16] Ghaheri A, Shoar S, Naderan M, Hoseini SS (2015). The Applications of Genetic Algorithms in Medicine. Oman Med J.

[17] Steyerberg EW, Harrell FE (2016). Prediction models need appropriate internal, internal-external, and external validation. Journal of clinical epidemiology.

[18] Lopez J (2011). Internal and external validation of a model to predict adverse outcomes in patients with left-sided infective endocarditis. Heart.

[19] Li H, Lai L, Chen L, Lu C, Cai Q (2015). The Prediction in Computer Color Matching of Dentistry Based on GA + BP Neural Network. Computational and mathematical methods in medicine.

[20] Liu R (2014). Bitterness intensity prediction of berberine hydrochloride using an electronic tongue and a GA-BP neural network. Experimental and Therapeutic Medicine.

[21] Gu Q (2010). VKORC1-1639G > A, CYP2C9, EPHX1691A > G genotype, body weight, and age are important predictors for warfarin maintenance doses in patients with mechanical heart valve prostheses in southwest China. European journal of clinical pharmacology.

[22] Li D, Xu J, Shi Y (2013). Research progress in the study of anticoagulant and low anticoagulant standards after valvular disease. Chin J Clin Thorac Cardiovasc Surg.

[23] Whitlock RP, Sun JC, Fremes SE, Rubens FD, Teoh KH (2012). Antithrombotic and thrombolytic therapy for valvular disease: Antithrombotic Therapy and Prevention of Thrombosis, 9th ed: American College of Chest Physicians Evidence-Based Clinical Practice Guidelines. Chest.

[24] Kimura M (2001). Effect of low-intensity warfarin therapy on left atrial thrombus resolution in patients with nonvalvular atrial fibrillation: a transesophageal echocardiographic study. Japanese circulation journal.

[25] Limdi NA (2009). Kidney Function Influences Warfarin Responsiveness and Hemorrhagic Complications. Journal of the American Society of Nephrology: JASN.

[26] Abou-Zied OK (2015). Understanding the physical and chemical nature of the warfarin drug binding site in human serum albumin: experimental and theoretical studies. Curr Pharm Des.

[27] Kucuk M (2016). Risk Factors for Thrombosis, Overshunting and Death in Infants after Modified Blalock-Taussig Shunt. Acta Cardiologica Sinica.

[28] Li D (2011). Low-intensity anticoagulation therapy in the pregnant women with mechanical heart valves: a report with 56 cases (Article in Chinese). Chinese Journal of Thoracic and Cardiovascular Surgery.

[29] Li D, Yingkong S, Zipu T, Xuzhong H, Hongshen Y (2001). The follow-up of 12 pregnant women with anticoagulation therapy after mechanical heart valve replacement (Article in Chinese). Chin J Obstet Gynecol.

[30] Lip GYH (2017). Anticoagulation Control in Warfarin-Treated Patients Undergoing Cardioversion of Atrial Fibrillation (from the Edoxaban Versus Enoxaparin-Warfarin in Patients Undergoing Cardioversion of Atrial Fibrillation Trial). The American journal of cardiology.

[31] Yu L-P, Song H-T, Zeng Z-Y, Wang Q-M, Qiu H-F (2012). Validation and comparison of pharmacogenetics-based warfarin dosing algorithms in Han Chinese patients. Zhonghua xin xue guan bing za zhi.

[32] Wei Z (2013). Large sample size, wide variant spectrum, and advanced machine-learning technique boost risk prediction for inflammatory bowel disease. American journal of human genetics.

[33] Li, Y. Guide Stable Warfarin Dose in Chinese Patients with Heart Valvular Replacement using Fu-Hua2 algorithm-Clinical medication accuracy study (Article in Chinese). *Chinese academy of medical sciences*, *Peking union medical college*. *2015* (2015).

[34] Mega JL (2015). Genetics and the clinical response to warfarin and edoxaban: findings from the randomised, double-blind ENGAGE AF-TIMI 48 trial. The Lancet.

[35] Sharabiani A, Bress A, Douzali E, Darabi H (2015). Revisiting Warfarin Dosing Using Machine Learning Techniques. Computational and mathematical methods in medicine.

[36] Groszek B, Piszczek P (2015). Vitamin K antagonists overdose. Przeglad lekarski.

[37] Heffler E, Campisi R, Ferri S, Crimi N (2016). A Bloody Mess: An Unusual Case of Diffuse Alveolar Hemorrhage Because of Warfarin Overdose. Am J Ther.

[38] Geçmen Ç, Kahyaoglu M, Yanýk E, Karatas MA, Izgi IA (2016). Massive liver hematoma secondary to overdose of warfarin treatment. Archives of the Turkish Society of Cardiology.

[39] Toker I, Duman Atilla O, Yesilaras M, Ursavas B (2014). Retropharyngeal Hematoma due to Oral Warfarin Usage. Turkish journal of emergency medicine.

[40] Levine M, Pizon AF, Padilla-Jones A, Ruha AM (2014). Warfarin overdose: a 25-year experience. Journal of medical toxicology: official journal of the American College of Medical Toxicology.

[41] Scott, R., Kersten, B., Basior, J. & Nadler, M. Evaluation of Fixed-Dose Four-Factor Prothrombin Complex Concentrate for Emergent Warfarin Reversal in Patients with Intracranial Hemorrhage. *The Journal of emergency medicine*, 10.1016/j.jemermed.2018.01.030 (2018). 10.1016/j.jemermed.2018.01.030 29510892

[42] Wang SV (2016). Prediction of rates of thromboembolic and major bleeding outcomes with dabigatran or warfarin among patients with atrial fibrillation: new initiator cohort study. BMJ (Clinical research ed.).

[43] Weitz JI, Harenberg J (2017). New developments in anticoagulants: Past, present and future. Thrombosis and haemostasis.

